# Effectiveness of Text Messaging Interventions on Blood Pressure Control Among Patients With Hypertension: Systematic Review of Randomized Controlled Trials

**DOI:** 10.2196/24527

**Published:** 2021-09-22

**Authors:** Hon Lon Tam, Eliza Mi Ling Wong, Kin Cheung, Siu Fung Chung

**Affiliations:** 1 School of Nursing Hong Kong Polytechnic University Hong Kong China (Hong Kong); 2 Kiang Wu Nursing College of Macau Macau Macao; 3 School of Nursing Tung Wah College Hong Kong China (Hong Kong)

**Keywords:** text messaging, hypertension, blood pressure, mHealth, meta-analysis

## Abstract

**Background:**

Controlling blood pressure (BP) is an international health concern, and high BP is a major contributor to cardiovascular disease mortality. Evidence has shown that educational interventions directed at patients potentially improve BP control and adherence to medications and lifestyle modifications. In addition, a text messaging intervention has a potential effect on BP control; however, the dosage of a text messaging intervention has not been determined in previous reviews, resulting in difficult application in practice.

**Objective:**

This review aimed to identify the effectiveness of a text messaging intervention on hypertension management with a specific focus on the dosage of text messaging and the type of additional interventions with text messaging.

**Methods:**

A systematic review was conducted and reported on in accordance with PRISMA guideline. Participants were aged 18 years and older and diagnosed with primary hypertension. The included studies used text messaging as a component of the intervention. We searched for randomized controlled trials published until June 30, 2020, from the following health-related electronic databases: Embase, Medline, CINAHL Complete, PsycINFO, and Scopus. Data were extracted for qualitative synthesis and meta-analysis. The Physiotherapy Evidence Database Scale was used to assess the methodological quality of each study, and the quality of the included studies was assessed independently by two authors.

**Results:**

Twelve studies met the inclusion criteria. The overall methodological quality was fair (mean score 5.75). The frequency of text message delivery varied from daily to biweekly. Health education was identified in 4 studies as an additional intervention with text messaging. The overall results showed that the text messaging intervention significantly reduced systolic BP (SBP) but not diastolic BP (DBP). There was no significant difference in BP reduction between studies that lasted 6 months or less and those that lasted more than 7 months. Seven studies that lasted 6 months or less involving 1428 patients with hypertension were pooled for further meta-analysis. Text messages delivered at a lower frequency (once per week or less) had a small effect on SBP reduction (effect size 0.35, *P*<.01) and DBP reduction (effect size 0.28, *P*=.01). In addition, the use of a text messaging intervention halved the odds of uncontrolled BP among patients with hypertension in 6 months (odds ratio 0.46, *P*=.02).

**Conclusions:**

This review found that a text messaging intervention was effective in BP control. One-way text messaging delivered in a weekly manner was suggested to be effective and required fewer resources. Future studies should use different forms of text message and be integrated into other interventions to improve adherence behaviors and BP control among patients with hypertension.

## Introduction

### Background

For a decade, hypertension (HTN) has been the leading risk factor for global disease burden [1]. Over 1 billion people are estimated to have HTN worldwide [2], which necessitates the development of national and international guidelines that provide scientific evidence for the control of blood pressure (BP) [3-8]. Persistent adherence to medications and lifestyle modifications is emphasized in the guidelines to control BP effectively; however, a low adherence rate has been noted in reviews and studies. Two systematic reviews have revealed that medication adherence among patients with HTN is 55% [9,10]. Regarding adherence to lifestyle modifications, studies have shown that adherence to one component of lifestyle modifications ranges from 14% to 85% [11-14], while only 1.7% to 23.6% of patients with HTN adhered to all components of lifestyle modifications: smoking cessation, limited alcohol consumption, regular exercise, maintenance of optimal body weight, and healthy diet [14,15].

Different interventions have been developed such as self BP monitoring, educational interventions, and health professional–led care to improve adherence to HTN management. Reviews suggested that educational interventions directed at patients not only improved BP significantly [16] but also had a significant effect on adherence to medications and lifestyle modifications [17,18]. The reviews further indicated that adding digital components such as text messaging potentially enhances the effect of educational interventions, resulting in improved BP control.

### Text Messaging Intervention

Systematic reviews of the effect of text messaging intervention on HTN management were not lacking; nonetheless, the results were inconclusive. Vargas and colleagues [19] included both quasi-experimental and randomized controlled trials (RCTs) in the review. They searched for articles published until July 2015, and 6 studies were included in the meta-analysis. Although the review revealed that the use of 2-month to 12-month text messaging interventions potentially decreases both systolic blood pressure (SBP) and diastolic blood pressure (DBP), the effect size was not determined because of insufficient data and high heterogeneity was noted because of different study designs [19]. In another systematic review, Islam et al [20] included RCTs published between January 1990 and July 2016, and the interventions lasted at least 6 months, with 70% of participants with cardiovascular diseases completing the study. Nine studies were included in the meta-analysis, and the results showed that interventions using a 6-month to 12-month text messaging intervention significantly reduced SBP and DBP among patients with cardiovascular diseases; however, high heterogeneity was observed as different dosages of text messaging intervention were pooled for meta-analysis [20]. Kassavou and Sutton [21] reviewed the use of voice messaging and text messaging interventions on medication adherence among patients with cardiometabolic diseases. They searched for RCTs published between January 1992 and April 2016. Of the 17 included studies, 9 used text messaging. The results of the meta-analysis indicated that the 25-day to 12-month interventions could improve medication adherence significantly; however, high heterogeneity was noted again because of wide variety of interventions used with text messaging. In addition, the authors did not differentiate the effects of text messaging from those of voice messaging on medication adherence [21]. Although the use of a text messaging intervention potentially improves BP control and medication adherence, the frequency of using a text messaging intervention varies from multiple messages per day to fewer than one message per week [19-21]. A review suggested that the use of 2-way text messaging, which required the participants to reply to the received text message, could improve BP control [19]. However, this suggestion was not based on the effect of pooled data. Thus, the directionality of effective text messaging remains inconclusive.

Other than a text messaging intervention, the use of smartphone apps and websites was reviewed and found to have a significant effect on BP control [22]. The interventions required participants to download a HTN smartphone app or access specific websites for HTN management. The ownership of a smartphone is the basic requirement for smartphone apps, and internet access is a prerequisite for both the apps and websites. However, concerns regarding the required technological competency and data protection have been raised [23]. Alternatively, a text messaging intervention can be delivered to recipients via a telecommunication network without any specific apps or internet access. Text message is compatible with being delivered and received between mobile phones and smartphones. Thus, the use of a test messaging intervention potentially covers more people than that of smartphone apps or websites.

### Research Gap

In summary, a text messaging intervention may improve HTN management; however, effective additional interventions with text messages have not been identified in previous reviews. A significant effect on BP reduction and medication adherence on HTN-related diseases could be noted if the intervention lasted more than 6 months, but the effectiveness of text messaging interventions lasting 6 months or less was unclear. Also, the dosage of text messaging in terms of frequency and directionality was inconclusive in previous reviews. Regarding the inclusion and exclusion criteria of previous reviews, the included studies were published until July 2016, which might not have reflected the increased use of mobile phones in recent years. As a text messaging intervention is simple to use and widely accepted by both mobile phone and smartphone users, it is worth including recent evidence to review the use of text messaging in HTN management.

### Aims

This review aimed to identify the effectiveness of a text messaging intervention in HTN management, with a specific focus on the dosage of text messaging interventions lasting 6 months or less in terms of frequency and directionality and the type of additional interventions with text messages.

## Methods

### Study Design

A systematic review and meta-analysis were conducted and reported in accordance with the Preferred Reporting Items for Systematic Reviews and Meta-analyses (PRISMA) statement [24], and the search was guided by the PRISMA framework:

- Participants: adults with HTN
- Interventions: text messaging
- Comparisons: standard care or usual care
- Outcomes: BP
- Study design: RCT

### Search Strategy

Keywords used in the search included hypertension, high blood pressure, adult, text messaging, text message, sms, short message service, and texting. The electronic databases related to health sciences used were Embase, Medline, CINAHL Complete (via EbscoHost), PsycINFO, and Scopus. Since the number of included studies in the previous review of the use of text messaging interventions was small [19-21], we searched for articles available in the databases until June 30, 2020 for screening. A manual search was conducted to identify potentially eligible studies from the reference lists of previous reviews [19-21].

### Selection Criteria

#### Participants

Adults aged 18 years and over who had been diagnosed with primary HTN were included. Patients diagnosed with secondary HTN or pregnancy-related HTN were excluded as they required different management strategies [3,8].

#### Interventions

Studies that used text messaging as a single or combined intervention were included. Studies using any specific HTN smartphone apps were excluded.

#### Comparisons and Design

Standard care or usual care provided to the control group was set as a comparator. For the 3-arm RCTs, a comparison was made between the control group and the text messaging intervention group. The comparison between the text messaging intervention group and other active intervention group was not conducted. The study design was limited to RCT to yield reliable evidence on the effectiveness of the interventions. All unpublished theses, conference papers, and non-English articles were excluded.

#### Outcome

The mean and standard deviation of both SBP and DBP were used as continuous data for the meta-analysis. The number of participants with uncontrolled BP in each group was used as dichotomous data for the meta-analysis.

#### Quality Assessment

The methodological quality of the RCTs was assessed using the Physiotherapy Evidence Database (PEDro) scale. The scale was designed to assess the quality of an RCT and assesses 11 items including randomization, allocation concealment, blinding, treatment of data, and dropout rate [25]. Among the items, 1 point was awarded to each item if the criteria were fulfilled; 10 of the items were rated, resulting in a total score range of 0-10. The total score <4 was considered low quality, which affected the applicability of the evidence [26]. A cutoff of 4 was used to determine the selection of each study. The quality of the RCTs was assessed independently by the first and fourth authors, and the disagreements were discussed and resolved by the second author.

### Data Synthesis and Meta-analysis

Data from eligible studies were extracted into a form containing the following information: authors, year of publication, type of RCT (2-arm or 3-arm), guiding theory, age of participants, setting, dosage of text messaging intervention, and outcomes.

Review Manager 5.4 (Cochrane Collaboration) was used in the meta-analysis to extract data regarding changes in SBP and DBP between baseline and final assessment. In accordance with the aim of this review, 3 subgroup analyses were conducted according to directionality, frequency, and combined interventions. Standardized mean difference (SMD) with a 95% confidence interval was used for the pooled effect of continuous data, SBP, and DBP. The *I*^2^ statistic was used to detect heterogeneity. A random effects model was used as it allows for different true effect sizes across studies [27]. SMDs equal to 0.2, 0.5, and 0.8 represented small, moderate, and large effects, respectively [28]. Regarding the dichotomous data on BP control, the number of participants with uncontrolled BP and total sample size in each of the intervention and comparator groups were used to determine the effect size and reported as odds ratio (OR). A sensitivity analysis was conducted to explore possible differences between the intention-to-treat analysis and others.

## Results

### Search Results

**Figure 1:**
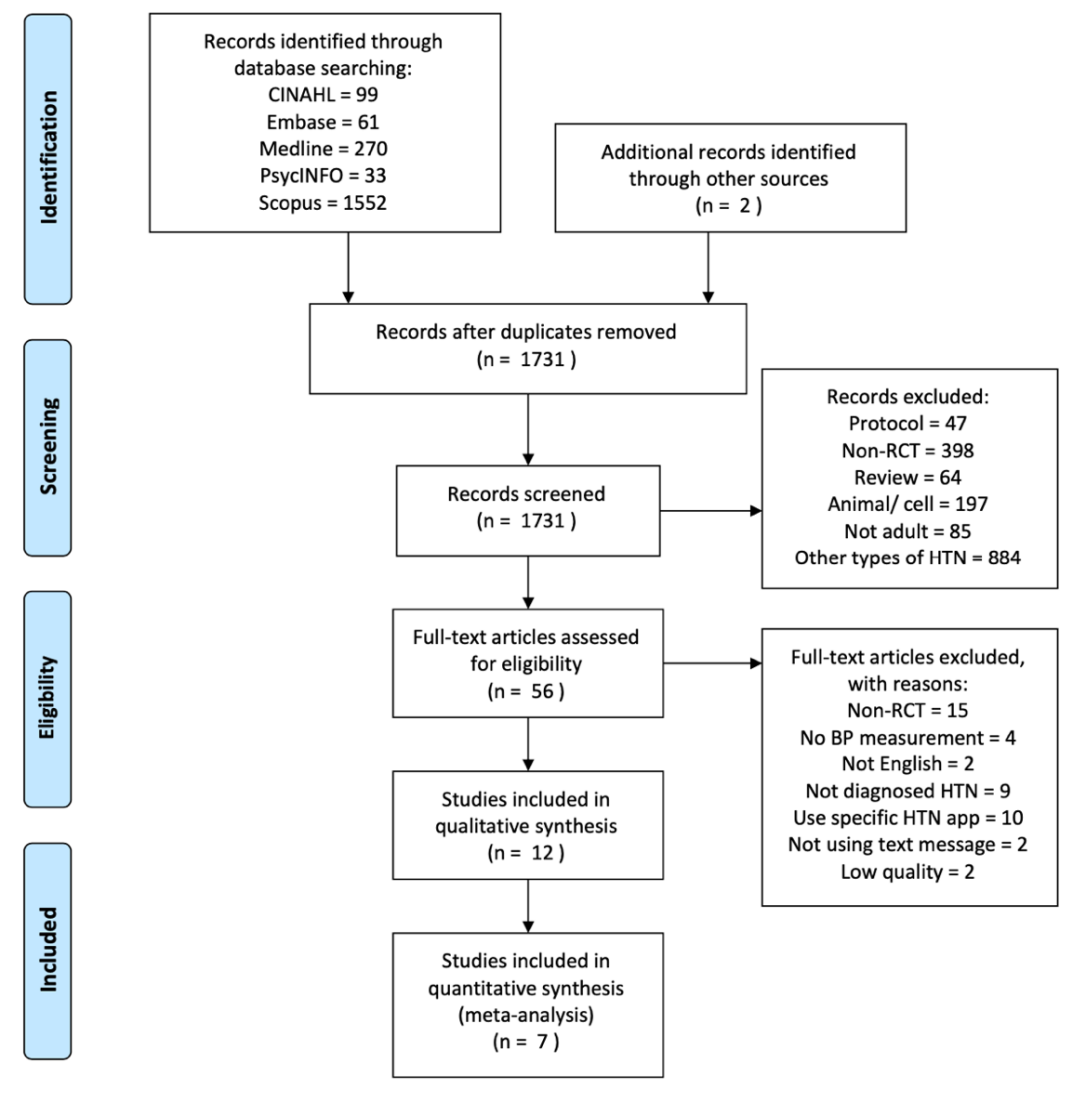
Flow diagram of literature selection process. BP: blood pressure; HTN: hypertension; RCT: randomized controlled trial.

A total of 1731 articles were initially identified from database searches, and 2 additional articles were identified in the manual search, as shown in Figure 1. A web-based application for systematic reviews, Rayyan, was used to remove duplicates and screen titles and abstracts [29]. Among the 56 articles assessed in full text, 2 were excluded due to low quality (PEDro score <4). The reasons for excluding those studies were that they did not conceal the group allocation process from the participants, therapists, and assessors; furthermore, only the participants from the intervention group withdrew from the study, and the data management and analysis methods were not stated clearly. Finally, 12 studies met the selection criteria. The characteristics of the studies and dosage of the text messaging intervention are described below.

### Quality of Studies

**Figure 2:**
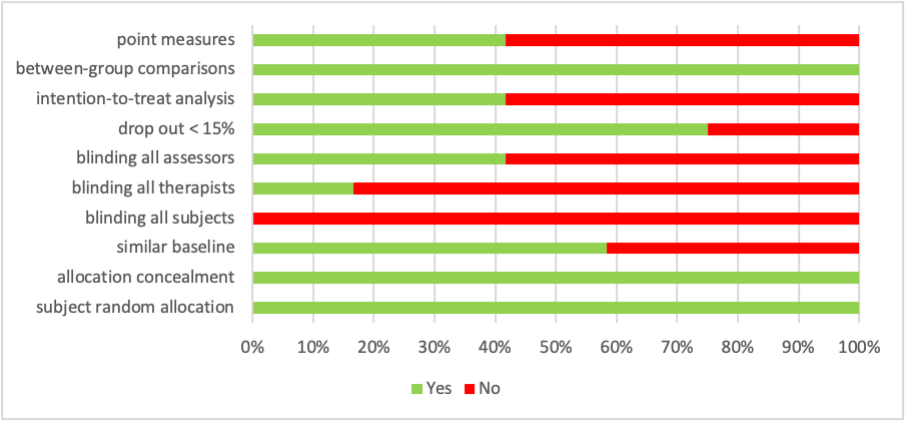
Methodological quality as percentage across all included studies.

Of the 12 included studies, the overall methodological quality was fair, and the PEDro score ranged from 4 to 8, with a mean score of 5.75 (Table 1). None of the studies was able to fulfill the participant-blinding criterion due to the nature of the text messaging intervention (Figure 2). Participants automatically knew their group allocation because no text messages were delivered to the control group. In addition, blinding of therapists was lacking in most studies in that the therapists knew the participant group allocation when providing care. The assessors in some studies knew the group allocation during follow-up data collection. Regarding the treatment of data, intention-to-treat was not followed in some studies, and clinical significance in each group was not assessed.

### Characteristics of Studies

The included studies were published between 2004 and 2020; 9 were published after 2016 [30-38]. The characteristics of the included studies are summarized in Table 1. Of the 12 included studies, 2 were 3-arm RCTs [34,39], and 4 studies provided the interventions lasted more than 7 months [31,36,37,39]. The number of participants in each study varied from 67 to 1432, with a mean age of 58.97 years. Participants mean age was over 60 years in 3 studies [32,38,40]. Four studies were conducted in the United States; 2 each in China and Spain; and one each in Argentina, South Africa, Chile, and Finland.

Regarding the additional interventions with text messaging, 4 studies integrated face-to-face health education with text messaging intervention to reinforce the effect on HTN management [31,33,38,41]. Only 4 studies were guided by theory, 2 of which used the health belief model [30,33], with the social cognitive theory and information-motivation-behavioral skills model used in 2 other studies [32,37].

**Table 1:** Characteristics of included studies.

Author	Duration of intervention (study design)	Frequency (directionality)	Theory	Additional intervention	PEDro^a^ score
Márquez Contreras et al [41]	6 m^b^ (2-arm)	2/w^c^ (1-way)	No	Health education, print materials	5
Carrasco et al [40]	6 m (2-arm)	4/w (1-way)	No	No	6
Bobrow et al [39]	12 m (3-arm)	1/w (1-way & 2-way)	No	No	8
Buis et al [30]	1 m (2-arm)	1/d^d^ (1-way)	Health belief model	No	4
He et al [31]	18 m (2-arm)	1/w (1-way)	No	Health education	6
Varleta et al [32]	6 m (2-arm)	1/14 days (1-way)	Social cognitive theory	No	5
Wan et al [33]	3 m (2-arm)	1/w (1-way)	Health belief model	Health education, phone call, booklet	7
Mehta et al [34]	4 m (3-arm)	1/d (2-way)	No	No	7
Zahr et al [35]	6 m (2-arm)	2/d (2-way)	No	No	4
Schroeder et al [36]	12 m (2-arm)	1/w (1-way)	No	No	7
Tahkola et al [37]	12 m (2-arm)	Varied (1-way)	Information-motivation-behavioral skills model	No	5
Zhai et al [38]	3 m (2-arm)	1/3 d (1-way)	No	Health education	5

^a^PEDro: Physiotherapy Evidence Database.

^b^m: month.

^c^w: week.

^d^d: day.

### Dosage of Text Messaging Interventions

The frequency of delivering text messages to patients with HTN varied from daily to biweekly among the included studies. Only one study used an individualized frequency to deliver text messages to the participants [37]. Text messages were delivered every day for the first 2 weeks, after which the frequency decreased from the third week onward. The first telephone follow-up was conducted in the fourth week to evaluate the participants’ BP levels. The frequency of text messages would increase thereafter if BP was not controlled at the follow-up. In addition, He et al [31] and Tahkola et al [37] tailored the content of text messages in accordance with the barriers identified in the assessment and follow-up.

Regarding the directionality, 2-way text messaging was seldom used in the studies. Zahr et al [35] required the participants to report SBP and DBP via the text messages. In the Mehta et al [34] 3-arm RCT, one intervention arm received an electronic pill box that was connected to an internet platform to monitor the use of medication, while another intervention arm required the participants to respond to text messages if they had taken the medication. The Bobrow et al [39] study was a 3-arm RCT that compared the effects of 1-way and 2-way text messaging on HTN management with the control group. Participants replied to text messages to change the medical appointments, delivery times, and language of the text message delivery. In comparison with the control group, the results of the study by Bobrow et al [39] showed that the use of 1-way text messaging decreased SBP significantly, whereas the decrease in SBP in the 2-way text messaging arm failed to achieve statistical significance. Bobrow et al [39] suggested that the nonsignificant findings might have been related to the older age of the participants or their experience in using technology.

### Other Outcomes

In addition to BP measurement, medication adherence was commonly assessed (Table 2). However, the methods of assessment differed among the studies. Six studies used self-reported measures to assess the medication adherence [30-33,36,38]. One study compared medication adherence between electronic pill boxes and text message responses [34] and another used the pill count [39]. Among the studies using self-reported measures, the Morisky Medication Adherence Scale was used in 3 studies. Most studies demonstrated an improvement in medication adherence after the use of text messaging. However, the diverse assessment methods resulted in difficulty in comparing medication adherence between studies. It is noteworthy that only one study used a self-reported approach to assess the adherence to lifestyle modifications among the patients with HTN [33].

**Table 2:** Outcome measures of included studies.

Author	Blood pressure	Medication adherence	Adherence to lifestyle modifications	Appointment adherence
Márquez Contreras et al [41]	+^a^	N^b^	N	–^c^
Carrasco et al [40]	–	N	N	N
Bobrow et al [39]	+	–	N	N
Buis et al [30]	–	–	N	N
He et al [31]	+	+	N	N
Varleta et al [32]	–	+	N	N
Wan et al [33]	+	+	+	N
Mehta et al [34]	–	–	N	N
Zahr et al [35]	–	N	N	N
Schroeder et al [36]	–	–	N	–
Tahkola et al [37]	–	N	N	N
Zhai et al [38]	+	+	N	N

^a^+: significant differences between intervention group (text messaging alone or with additional interventions) and control group (standard care or usual care; *P*<.05).

^b^N: not measured.

^c^–: nonsignificant differences between intervention and control groups.

### Meta-analysis

**Figure 3:**
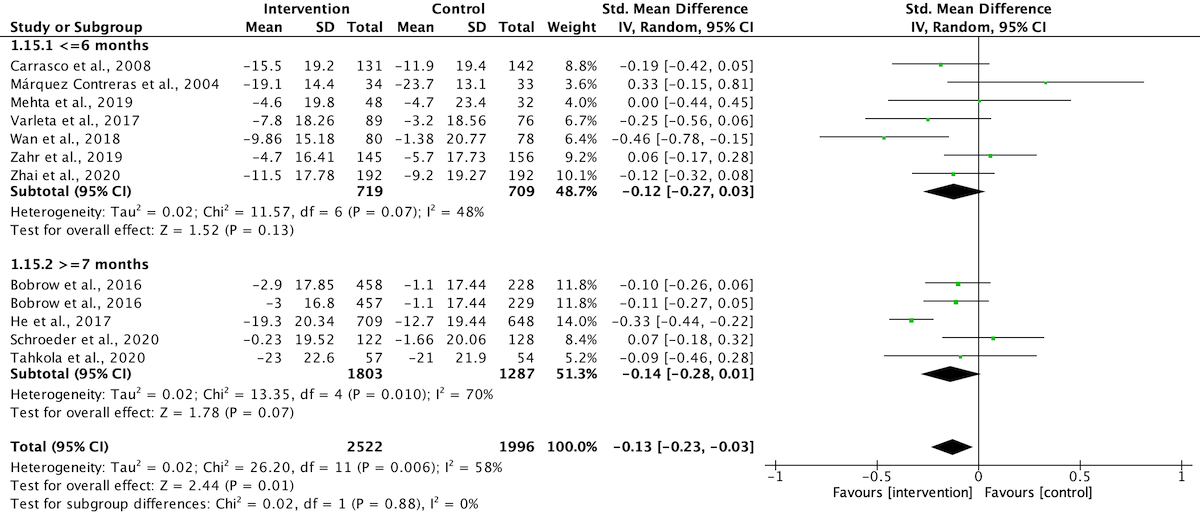
The effect of study duration on systolic blood pressure reduction.

**Figure 4:**
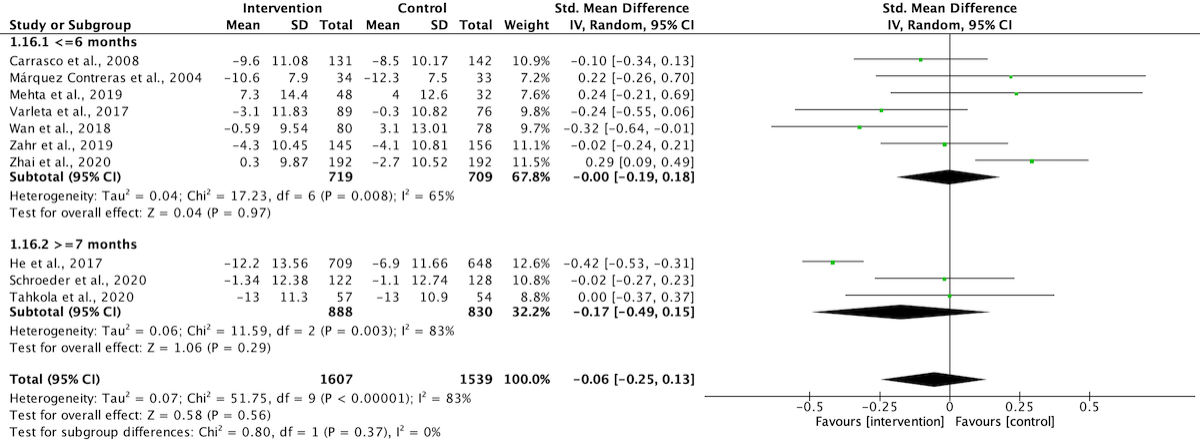
The effect of study duration on diastolic blood pressure reduction.

Four studies used interventions that lasted 12 months or more, while the interventions in 8 studies lasted 6 months or less. The corresponding authors of 2 studies were contacted via email to obtain unreported data, but only 1 of them provided the requested data [32]. The study from which unreported data could not be obtained was excluded from the meta-analysis. In the Bobrow et al [39] 3-arm RCT, text messaging was used in 2 intervention groups, and 2 comparisons with control group were extracted for meta-analysis. In the Mehta et al [34] 3-arm RCT, text messaging was used in one intervention group, while an electronic pill box was used in another intervention group. Thus, only one comparison was performed. Although some long-term studies, such as those by Bobrow et al [39] and He et al [31], provided midprocess data at 6 months, the interventions were not delivered to completion, and the 6-month data failed to reflect the holistic effect of the interventions. The midprocess data at 6 months was not extracted for meta-analysis. Figures 3 and 4 illustrate the durations of intervention lasting 6 months or less and those lasting more than 7 months made no statistical differences in SBP and DBP reduction (subgroup differences, *P*>.05). The overall results revealed that the text messaging intervention significantly reduced SBP (Figure 3, SMD=.13, *P*=.01) but not DBP (Figure 4, SMD=.06, *P*=.56). To explore the effectiveness of text messaging interventions lasting 6 months or less, we excluded all studies with interventions lasted more than 7 months. Also, the study without obtaining unreported data was excluded. As a result, 7 studies lasting 6 months or less were pooled for meta-analysis in terms of directionality, frequency, and type of intervention.

**Figure 5:**
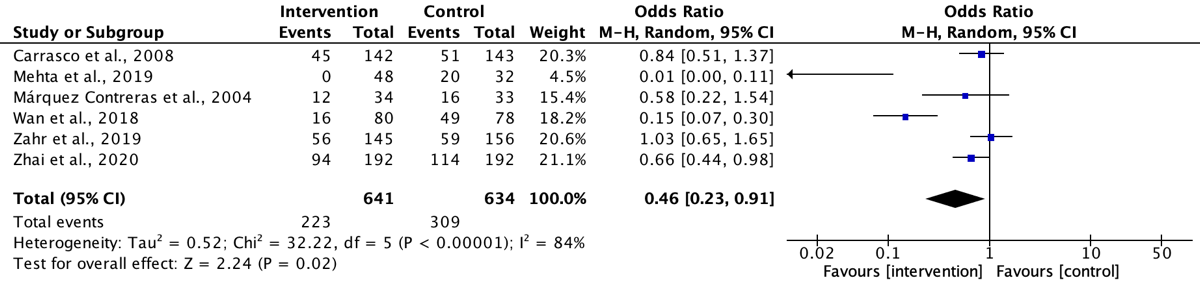
Odds ratio of text messaging on blood pressure control.

Tables 3 and 4 show the effectiveness of a text messaging intervention on SBP and DBP reduction with interventions lasted 6 months or less. Regarding the directionality of text messaging, neither 1-way nor 2-way text messaging had a significant effect on SBP or DBP reduction. However, a small effect on SBP and DBP reduction was noted when text messages were delivered in a weekly manner (ie, 1 text message per week). However, the use of a text messaging intervention alone or in combination with health education did not significantly affect SBP or DBP reduction. Six studies reported the number of participants with uncontrolled BP (SBP ≥140 mm Hg or DBP ≥90 mm Hg) at the end of the study. The data were pooled as shown in Figure 5, and the use of a text messaging intervention helped patients with HTN achieve a controlled BP with SBP <140 mm Hg and DBP <90 mm Hg (OR 0.46, *P*=.02).

**Table 3:** Effectiveness of text messaging on systolic blood pressure reduction with interventions lasting 6 months or less.

Subgroup analysis			Number of studies		Effect size (95% CI)		Heterogeneity *I*^2^ (%)		*P* value		Significance of subgroup differences (*P* value)	
**Directionality of text messaging**												.10
	1-way	5		0.18 (0.00, 0.36)		49		.05		—^a^		
	2-way	2		–0.05 (–0.25, 0.15)		0		.65		—		
**Frequency of text messaging**												.02
	>1 per week	5		0.04 (–0.09, 0.18)		22		.53		—		
	≤1 per week	2		0.35 (0.13, 0.57)		0		.002		—		
**Type of intervention**												.87
	With health education	3		0.13 (–0.23, 0.48)		74		.49		—		
	Text messaging only	4		0.09 (–0.06, 0.24)		14		.23		—		

^a^Not applicable.

**Table 4:** Effectiveness of text messaging on diastolic blood pressure reduction with interventions lasting 6 months or less.

Subgroup analysis		Number of studies	Effect size (95% CI)	Heterogeneity *I*^2^ (%)	*P* value	Significance of subgroup differences (*P* value)	
**Directionality of text messaging**							.58
	1-way	5	0.03 (–0.22, 0.29)	75	.79	—^a^	
	2-way	2	–0.03 (–0.24, 0.17)	0	.75	—	
**Frequency of text messaging**							.01
	>1 per week	5	–0.10 (–0.28, 0.08)	50	.27	—	
	≤1 per week	2	0.28 (0.06, 0.50)	0	.01	—	
**Type of intervention**							.97
	With health education	3	–0.06 (–0.48, 0.36)	81	.77	—	
	Text messaging only	4	0.07 (–0.08, 0.21)	10	.37	—	

^a^Not applicable.

### Sensitivity Analysis

Among the 11 studies with sufficient data pooled in the meta-analysis, 5 used intention-to-treat analysis and the rest did not clearly state their method of analysis. No significant subgroup difference in SBP and DBP reduction was noted between the intention-to-treat analysis and others (Multimedia Appendix 1). Seven studies with text messaging interventions lasted 6 months or less, 2 of which used intention-to-treat analysis. Figure S4 showed a significant subgroup difference in DBP reduction (Multimedia Appendix 1). The pooled results of studies using intention-to-treat analysis showed a favorable effect in the control group, in which usual care reduced DBP more effectively than the text messaging intervention [34,38]. No significant subgroup difference in SBP reduction was noted among interventions that lasted 6 months or less (Multimedia Appendix 1).

## Discussion

### Summary

This review identified 12 RCTs from 5 electronic databases. The study duration varied from 1 to 18 months. Five studies were published between January 2019 and June 2020, showing that the interest in using text messaging has increased in HTN research. In the meta-analysis, the use of a text messaging intervention significantly reduced SBP but not DBP. Seven studies with a text messaging intervention lasting 6 months or less, involving 1428 patients with HTN, were pooled for further analysis. The results showed that the delivery of weekly text messages significantly improved both SBP and DBP. In addition, the use of a text messaging intervention halved the odds of uncontrolled BP in patients with HTN in 6 months. This review provided information regarding the dosage of a text messaging intervention and type of additional interventions in HTN research using text messages.

### Directionality and Frequency

A systematic review of patients with HTN claimed that the use of 2-way text messaging potentially reduces SBP and DBP [19]; however, our findings did not reveal any significant effect relating to the directionality of text messaging on SBP and DBP reduction with an intervention lasting 6 months or less. Noteworthily, participants were required to respond when 2-way text messaging was used. In terms of simple yes/no responses, a session on how to reply to text messages was necessary if specific information, such as BP level, was required. The use of 2-way text messaging may potentially require additional resources. Head et al [42] found that the use of 1-way text messaging significantly increased the likelihood of healthy behaviors. The Bobrow et al [39] 3-arm RCT also showed that the use of 1-way text messaging decreased SBP significantly. Having the same tendency, our findings revealed that 1-way text messaging might potentially reduce SBP (Table 3, SMD=.18, *P*=.05). Therefore, 1-way text messaging might be more feasible and effective for patients with HTN.

Regarding frequency, a decreasing frequency was found to have a moderate effect on health promotion in a review [42]. Since only 1 included study used a decreasing frequency of text messaging on HTN management [37], the effect was not examined in this review. Accordingly, a previous review revealed that the daily or weekly delivery of text messages had a minute effect on promoting health behaviors [42]. Our findings revealed that text messaging could reduce SBP as well as the odds of uncontrolled BP given a target population of patients with HTN and the weekly delivery of text messages.

### Types of Intervention

Health education was a commonly used intervention in combination with text messages among the included studies. In this review, a text messaging intervention alone and the combined use of health education had no significant effect on SBP and DBP reduction. The findings contradicted those of a recent review, in which the use of supportive methods, such as text messages and take-home reading materials, with health education yielded a moderately significant effect in improving both SBP and DBP among patients with HTN [18]. In addition, a review by Head et al [42] found that a text messaging intervention alone and the combination of text messages with other interventions could have a significantly small effect on health promotion; however, the duration of their included studies was not limited, and a wide variety of combined interventions was noted such as websites, health education, print materials, pedometers, and daily records of health behavior. Hence, the effectiveness of a text messaging intervention alone or in combination with other interventions to manage HTN remains inconclusive.

On the other hand, tailored content of text messages showed a significant effect on health promotion and medication adherence in previous reviews [21,42]. Two studies in this review were found to tailor their text messages to the participants [31,37], but the interventions lasted 12 to 18 months, resulting in an effect that was not analyzed in the meta-analysis. He et al [31] recruited 1432 patients with HTN in their study and revealed that the tailored content reduced SBP and DBP significantly; however, a significant reduction in SBP and DBP was not noted in the study by Tahkola et al [37]. Another study recruiting people with uncontrolled BP showed that tailored content did not make a significant difference in the reduction of SBP and DBP [43]. The effect of tailored content of text messages on SBP and DBP in patients with HTN was inconclusive. In addition, tailored content requires additional resources, as He et al [31] estimated that the use of tailored content would cost an extra US $6.36 per patient per month. Therefore, tailored content of text messages may not be beneficial to patients with HTN, especially in health care systems with limited resources.

### Assessment of Adherence to HTN Management

Medication adherence was assessed in most of the included studies. However, adherence to lifestyle modifications is the core element in HTN management guidelines for patients with HTN and those at risk of HTN [3-8]. The only included study that assessed adherence to lifestyle modifications was conducted by Wan et al [33] in 2018. They revealed that the use of standardized content of text messages combined with health education and leaflet intervention could improve adherence behaviors in 3 months [33]. Another 2-arm RCT recruited 710 patients with coronary heart diseases and showed that adherence to lifestyle modifications improved significantly when tailored content of text messages was delivered 4 times a week for 6 months [44]. These studies suggest that a text messaging intervention may be an effective method to improve adherence to lifestyle modifications. Future studies should examine the effect of text messaging on adherence to both medications and lifestyle modifications among patients with HTN. Regarding the choice of adherence scale, the Treatment Adherence Questionnaire for Patients with Hypertension [45] was suggested in 2 systematic reviews, as it is a comprehensive measure that covers adherence to both medication and lifestyle modifications and is designated for patients with HTN [46,47].

### Intention-to-Treat Analysis

A significant difference of DBP reduction in control group was found in the sensitivity analysis (Multimedia Appendix 1). The significant result was from studies that delivered text messages more frequently (more than once per week) [34,38]. The significant DBP reduction in the control group may indicate information overload by means of high frequency of text messaging [48].

### Limitations

Several limitations were noted in this review. We searched for and included only English articles, thus reducing the diversity of the analyzed studies. Only 7 studies were included in the subgroup analysis. Hence, the findings should be interpreted with caution, since the subgroup analyses consisted of a very small number of studies and the variations in dosage of text messaging and combination with health education caused high heterogeneity in the meta-analysis. The decision to only include RCTs in the meta-analysis increased the internal validity of the findings; nonetheless, the external validity might have decreased. The scope of the review was limited to the text messaging, and advanced features added to today’s mobile phones or smartwatches to enhance HTN management were not explored.

### Implications

We have provided advice for the use of text messaging in HTN management in practice and research. The use of text messaging could help patients with HTN return to controlled BP levels in 6 months or less, and 1-way text messaging could be useful if delivered weekly to patients with HTN. The standardized content of text messages can be stored in a preprogrammed database. Once clients agree to receive text messages, their mobile phone numbers can be entered into the program, thus facilitating the regular delivery of text messages to the clients.

The rapid growth of smartphone ownership and advancing technologies in recent decades has evolved a new mode of delivering text messages. Data from the Pew Research Center showed that smartphone ownership in advanced economies increased from 68% to 76% between 2015 and 2018 [49,50]. Instead of using a traditional short message service, text messages can now be delivered in the form of voice, images, and videos via general smartphone communication apps such as Telegram and WhatsApp. The advanced features of text messaging may enhance adherence behaviors, resulting in an improvement in BP control [51,52]. Hence, future studies should examine the effect of informative images and videos on HTN management, including BP regulation, medication adherence, and adherence to lifestyle modifications. The integration of text messaging with advanced technology should be explored in future studies.

### Conclusions

This review updates the information from previous reviews with a focus on patients with HTN. The meta-analysis provides evidence for the use of a text messaging intervention in BP control. As indicated in the meta-analysis, the use of a text messaging intervention lowers the odds of uncontrolled BP. Text messages delivered at a lower frequency had a small effect on the reduction of SBP and DBP. Although the effect of a text messaging intervention on medication adherence was not examined, most included studies showed improvement after the intervention. Thus, text messaging is a potentially useful intervention for HTN management. Regarding the implications of this review, weekly 1-way text messaging is recommended in practice and research. The use of text messages should be incorporated into different interventions in future studies to further improve adherence behaviors and BP control among patients with HTN. There is great potential for professional development in the area of using advanced features of text messages and the more feasible use of these features in delivering messages to clients effectively.

## Appendix

Multimedia Appendix 1 Sensitivity analysis.

## Abbreviations

BP: blood pressure
DBP: diastolic blood pressure
HTN: hypertension
OR: odds ratio
PEDro: Physiotherapy Evidence Database
PRISMA: Preferred Reporting Items for Systematic Reviews and Meta-analyses
RCT: randomized controlled trial
SBP: systolic blood pressure
SMD: standardized mean difference
