# Supplementary material for: Effectiveness of Text Messaging Interventions on Blood Pressure Control Among Patients With Hypertension: Systematic Review of Randomized Controlled Trials
Source: JMIR Mhealth Uhealth. 2021 Sep 22;9(9):e24527. doi: 10.2196/24527 (PMC8495578; doi:10.2196/24527)

Figure S1. Effect of statistical analysis on SBP reduction in all studies.


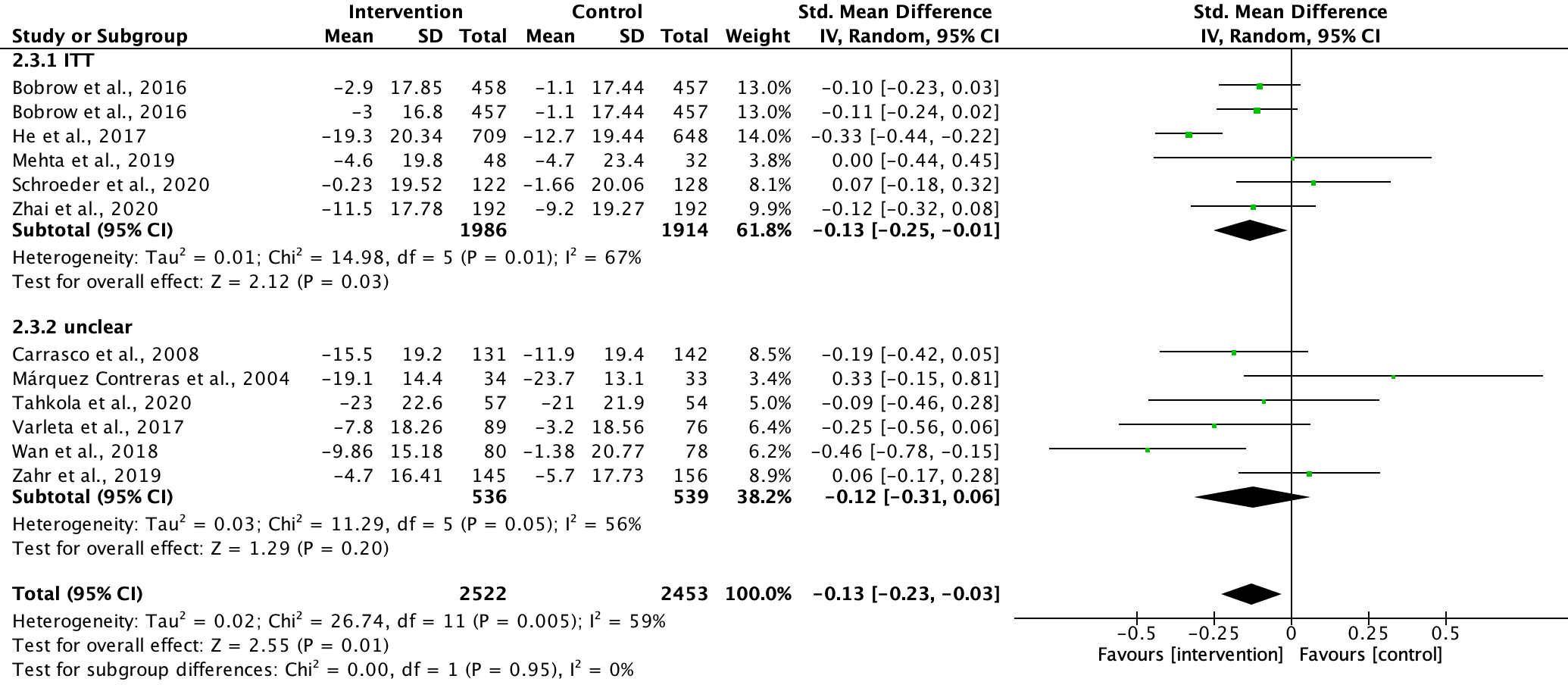


Figure S2. Effect of statistical analysis on DBP reduction in all studies.


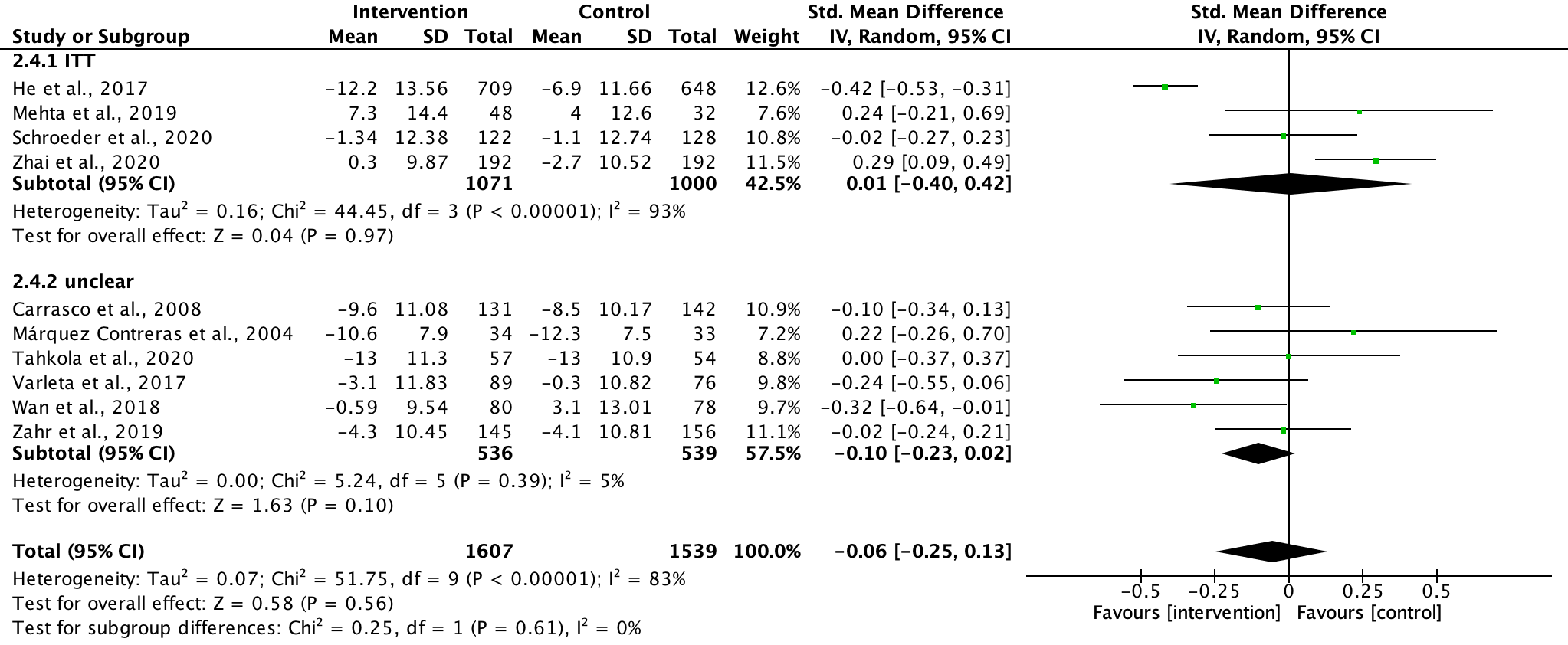


Figure S3. Effect of statistical analysis on SBP reduction in studies that lasted less than 6 months.


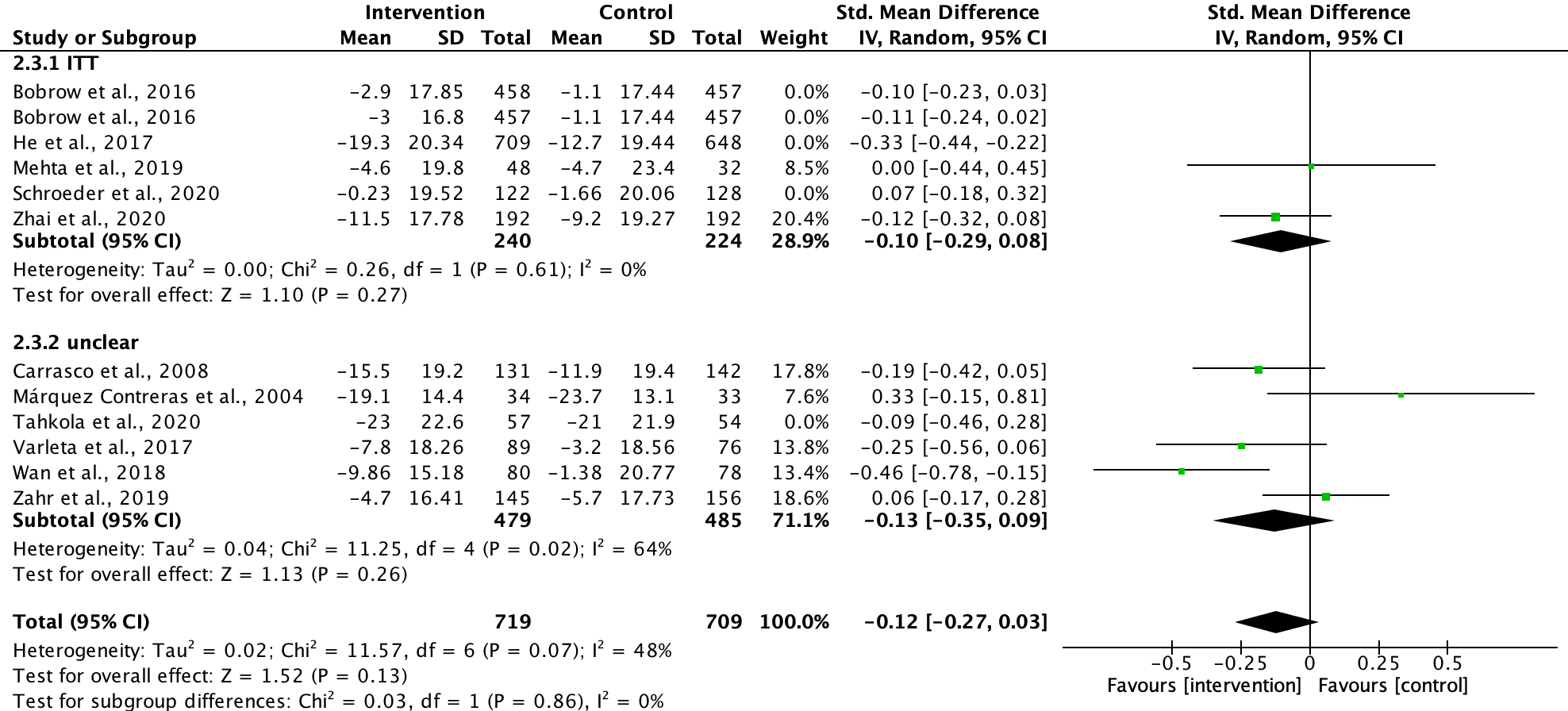


Figure S4. Effect of statistical analysis on DBP reduction in studies that lasted less than 6 months.


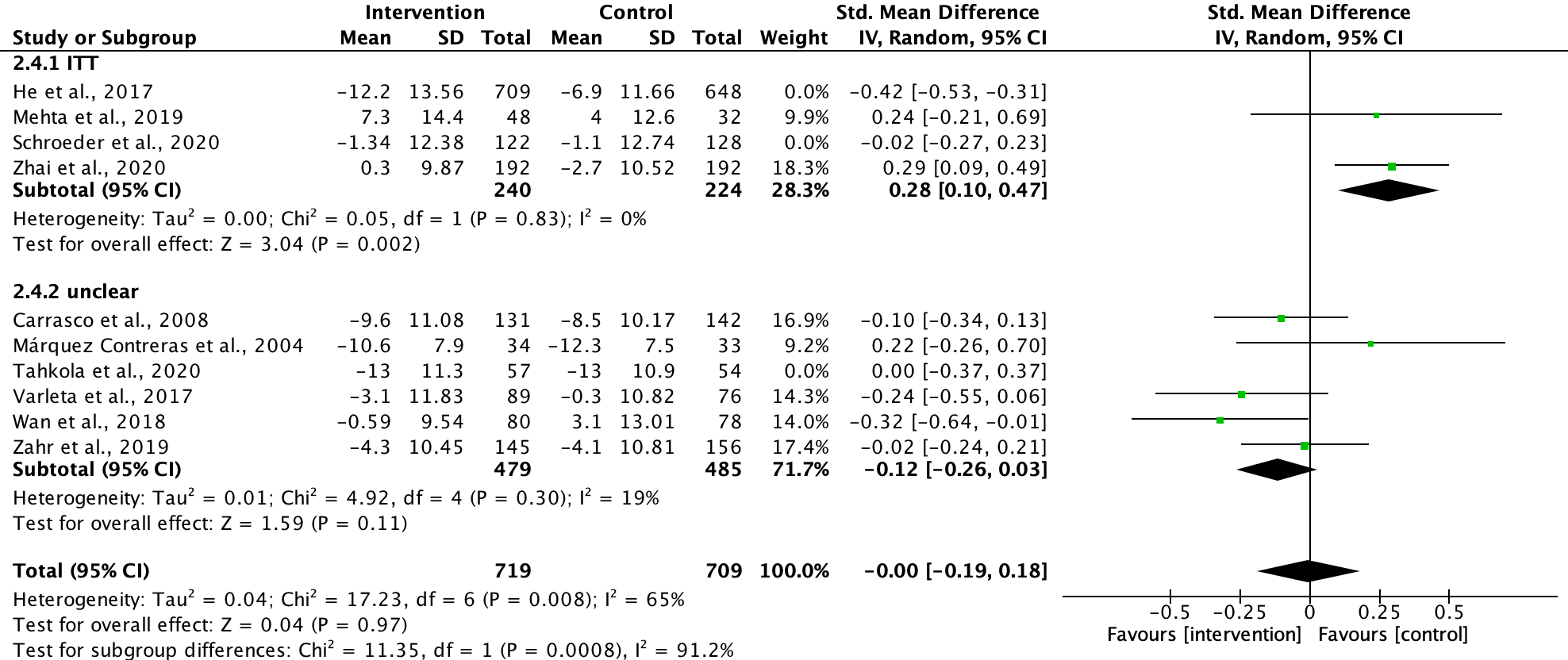

Supplement: Multimedia Appendix 1 [file mhealth_v9i9e24527_app1.doc]
